# Mental Health Client Experiences of Telehealth in Aotearoa New Zealand During the COVID-19 Pandemic: Lessons and Implications

**DOI:** 10.2196/47008

**Published:** 2023-05-26

**Authors:** Tara N Officer, Marika Tait, Karen McBride-Henry, Laura Burnet, Benjamin J Werkmeister

**Affiliations:** 1 School of Nursing, Midwifery, and Health Practice Te Herenga Waka - Victoria University of Wellington Wellington New Zealand; 2 School of Health Te Herenga Waka - Victoria University of Wellington Wellington New Zealand; 3 Department of Psychological Medicine University of Otago (Wellington) Wellington New Zealand

**Keywords:** telehealth, mental health service delivery, COVID-19, Aotearoa New Zealand, clients, patient-centered care, telemedicine, mental health, experience, satisfaction, perception, perspective, attitude

## Abstract

**Background:**

The COVID-19 pandemic and consequent lockdowns disrupted mental health service delivery worldwide, accelerating the adoption of telehealth services to provide care continuity. Telehealth-based research largely highlights the value of this service delivery method for a range of mental health conditions. However, only limited research exists exploring client perspectives of mental health services delivered via telehealth during the pandemic.

**Objective:**

This study aimed to increase understanding of the perspectives of mental health clients around services provided via telehealth over the 2020 COVID-19 lockdown in Aotearoa New Zealand.

**Methods:**

Interpretive description methodology underpinned this qualitative inquiry. Semistructured interviews were conducted with 21 individuals (15 clients and 7 support people; 1 person was both a client and support person) to explore their experiences of outpatient mental health care delivered via telehealth during the COVID-19 pandemic in Aotearoa New Zealand. A thematic analysis approach supported by field notes was used to analyze interview transcripts.

**Results:**

The findings reveal that mental health services delivered via telehealth differed from those provided in person and led some participants to feel they need to manage their own care more actively. Participants highlighted several factors affecting their telehealth journey. These included the importance of maintaining and building relationships with clinicians, the creation of safe spaces within client and clinician home environments, and clinician readiness in facilitating care for clients and their support people. Participants noted weaknesses in the ability of clients and clinicians to discern nonverbal cues during telehealth conversations. Participants also emphasized that telehealth was a viable option for service delivery but that the reason for telehealth consultations and the technicalities of service delivery needed to be addressed.

**Conclusions:**

Successful implementation requires ensuring solid relationship foundations between clients and clinicians. To safeguard minimum standards in delivering telehealth-based care, health professionals must ensure that the intent behind telehealth appointments is clearly articulated and documented for each person. In turn, health systems must ensure that health professionals have access to training and professional guidance to deliver effective telehealth consultations. Future research should aim to identify how therapeutic engagement with mental health services has changed, following a return to usual service delivery processes.

## Introduction

In March 2020, Aotearoa New Zealand went into the first of multiple lockdowns (similar to stay-in-place orders) to curtail COVID-19 transmission. People remained home, having little contact with those outside their household, and health care was limited to those receiving and delivering essential services. New Zealand’s response to COVID-19 was considered among the most stringent globally and successfully delayed COVID-19 community transmission [1,2].

Lockdowns had negative implications for some members of New Zealand’s population with preexisting mental health conditions [3,4]; in 1 survey, over half of those with a preexisting diagnosis experienced moderate or severe distress over lockdown [5]. Research into mental health presentations at Christchurch Hospital emergency department during the COVID-19 lockdowns indicated increased overdose and self-harm hospital presentations in the general NZ population [6], other research emphasized higher mental health ambulance callouts nationally [7], and negative impacts on well-being [8]. Mental health services, such as addiction support services, were in high demand managing increasingly complex cases but were unable to provide health care in the traditional in-person manner; instead, these services adopted telehealth [9], including telephone and videoconferencing.

There is a body of international research demonstrating the value of telehealth-based care for a variety of mental health conditions including during the pandemic [10-15], albeit with potential limitations related to evidence for types of telehealth-based therapy [14,16] and findings drawn from smaller studies with limited controlled trials [12,13]. There has also been growing international research into people’s perspectives on receiving telehealth-based mental health services over the pandemic for a variety of population groups, including those with dementia [17], youths [18], and the general adult population [19,20].

This study fills a gap in extant literature by investigating the experiences of clients receiving mental health treatment via telehealth in 3 New Zealand outpatient services over the 2020 COVID-19 lockdown. These services deliver care to the most high-risk clients; although ringfenced to offer care to the 3% of most severe at-risk clients, recent evidence suggests 5% of New Zealand’s population falls within this severe category [21]. The severity of client needs and resourcing issues mean that this service generally fails to meet targets for wait times, and service integration remains fragmented [22]. This research differs from other research in that it explores views of mental health clients irrespective of the condition being treated or the treating team. In this way, our research provides an in-depth understanding of the implications of implementing a telehealth service for a high-risk population. This is important within a service that has a well-documented history of underresourcing and underfunding [21] as it should help inform changes to service delivery and inform discussions of the implications of New Zealand’s stringent COVID-19 response.

## Methods

### Methodology

An interpretive description methodology [23] was used to guide research processes. This methodology facilitates iterative shared understanding from material such as interviews (as in this study) to formulate thematic descriptions to influence clinical practice [24].

### Ethics Approval

Ethics approval for this study was granted by the Te Herenga Waka—Victoria University of Wellington Human Ethics Committee (#28808) in August 2020. Following this, research endorsement was provided by the relevant health district Research Advisory Group (Māori) (#765). This group assessed the cultural suitability and appropriateness of the project for Indigenous Māori participants. Before engaging in this research, participants provided written informed consent. Protecting participant privacy and confidentially, participants were assigned unique identifiers (eg, “P5”); support people (parents, guardians, or caregivers) were identified with an appended “s” (eg, “P5s”). In addition, participants received a NZD $50 (~USD $30) voucher of their choice to thank them for their participation in our research.

### Study Location

This research was conducted with clients receiving services from one or more of 22 outpatient teams operating within 3 publicly funded mental health services. Team size varies from between 5 and 26 clinicians and includes a variety of professions such as doctors, nurses, clinical psychologists, social workers, occupational therapists, and cultural workers. Child and adolescent, and adult, mental health teams offer services to their geographic region; some services specifically target population groups such as Indigenous Māori and high-risk Pacific populations, and older adults.

Interviewing commenced in October 2020. The region had completed 1 lockdown at this time, and restrictions on gatherings and social distancing had largely been lifted. However, following interviews, the country and study region went through several additional lockdowns.

### Data Collection

Participant recruitment occurred initially via the team leaders of each outpatient team. Team leaders received an information sheet, consent form, and other information on recruiting clients. After initial contact with the outpatient team leader, and in cases of no response, up to 3 separate follow-ups (emails or telephone calls) were conducted. Of the 22 outpatient teams, 1 did not respond to any attempts at contact. However, in most cases, team leaders indicated that they wanted the research team to attend the team’s weekly meeting to introduce the study; in these cases, a combination of TNO, LB, and BJW attended these meetings.

The outpatient team leader or clinicians in the team then identified potential clients to participate in an interview. Clients had attended at least one telehealth-based outpatient appointment over the period of lockdown, had an established relationship with their team (ie, had received services from the team for at least 5 months), and were judged by the team to be sufficiently well to participate in an interview. Despite this judgment, owing to the nature of outpatient mental health, clients were likely to be considered as having the most severe need for mental health services. Where individuals were younger than 18 years, the client’s parent or guardian was instead invited to participate in the research.

The team leader or relevant clinicians then passed research details on to their clients, who could contact the research team via email, telephone, or web-based form. Sixteen clients contacted the research team, and 15 participated in semistructured interviews based on an interview schedule that had been pilot tested for length and sense (see Multimedia Appendices 1 and 2). Recruitment ceased once no new themes were emerging through the interviews, which is in keeping with interpretive description’s methodological boundaries that there was confidence that findings offered sufficient relevant variation and captured topic complexity [24].

Most interviews were conducted in person, with 1 conducted by telephone. Participants could bring support people with them to the interview. These people could offer their views on mental health service delivery over lockdown. Seven support people participated in interviews. In total, 21 people were interviewed as 1 person was both a client and a support person. Interviews were conducted by LB and TNO together, or by TNO, lasting between 19 and 69 minutes (median ~39.5 minutes). Field notes were completed following interviews.

Participants were also surveyed about their key demographic characteristics (age range, ethnicity, gender, and time working with outpatient mental health services), their access to telephone and internet, and familiarity with videoconferencing tools.

The research team comprised clinician-researchers (BJW, KMH, and TNO) and experts in education with a mental health focus (LB) and science communication (MT). BJW, a psychiatric registrar who worked within the mental health teams studied, did not conduct any interviews, and was kept blinded to the identity of participants but provided support in understanding how services were delivered within the study location. Much of the research team had lived experience of supporting people with mental health challenges.

### Data Analysis

This research followed an iterative and constant comparative process [25]; as new findings emerged throughout the thematic analysis, subsequent interviews facilitated the ability to gain further elaboration. In this way, research participants were given the opportunity to “result check” prior interviews. All interviews were transcribed by a third party and then checked for accuracy and completeness by LB. Following this, interview transcripts were returned to participants who requested them. Any changes participants made to transcripts were then included in the analyzed transcript.

Following discussions between BJW, LB, and TNO and agreement on an initial coding framework, LB firstly analyzed data using NVivo 12 (QSR International). TNO then iteratively reviewed and checked coding before BJW, LB, and TNO came together to discuss interpretations of findings to improve finding credibility.

## Results

### Overview

Most participants could access the telephone and internet; 3 had internet access only. Participants had a range of experiences with outpatient mental health services, and most used services intermittently for multiple years with a range of providers. Eight participants agreed or strongly agreed that they were very familiar with videoconferencing, and 9 participants disagreed or strongly disagreed about their familiarity with videoconferencing. All participants described in-person care as their usual form of service delivery, and all used a range of telehealth mediums (email, SMS text message, telephone, and videoconferencing) with some participants still having minimal in-person care for depot injections and physical monitoring. Participant characteristics are presented in Table 1; 1 support person did not answer demographic questions.

Six themes emerged from the interview analysis. One theme underscores general changes to service delivered by telehealth. The remaining five highlight intangible factors such as (1) maintaining and building relationships; (2) discerning nonverbal cues; (3) creating safe spaces and involving others; (4) collaboration and clinician readiness; and (5) having options.

**Table 1:** Participant characteristics.

Participant	Gender	Age range (years)	Ethnicity
P1	Male	35-44	NZ^a^ European
P2	Female	35-44	NZ European
P3s	Female	45-55	NZ European
P4	Gender diverse	18-25	Māori, NZ European
P5s	Male	35-44	NZ European
P6	Female	65+	Dutch
P7	Female	35-44	Māori
P7s	Male	Not reported	Not reported
P8	Female	18-25	Māori
P9	Female	55-64	NZ European
P10	Male	≥65	NZ European
P10s	Female	≥65	NZ European
P11	Male	35-44	NZ European
P12	Female	45-54	NZ European
P12s	Female	≥65	NZ European
P13	Female	55-64	NZ European
P13s^b^	Male	35-44	NZ European
P14	Female	35-44	Mixed ethnicity American
P15	Female	≥65	NZ European
P16	Female	25-34	NZ European
P17s	Female	45-54	NZ European

^a^NZ: New Zealand.

^b^Participant 13s was a mental health client and a support person.

### Changes to Service Delivered by Telehealth

Participants described receiving varied information on plans for service delivery over lockdown. Notably, all recognized a named clinician as their point of contact with the outpatient service. Some experienced a smooth transition to a telehealth-only service and received plenty of information. Others felt they received no information and little support.

Participants were generally easily able to contact clinicians and received the same or more frequent contact with their mental health clinicians during the lockdown. However, participants indicated that during lockdown the type of contact differed from usual; there were more check-ins via SMS text message or telephone rather than regular appointments or provision of clinical services. This led some to feel alone in managing their own care:

I ended up feeling more like I was the only one who could do anything for me. I felt, I don’t want to say abandoned but just that whole sense of isolation and it’s like they can’t do anything. It made me really feel very alone.

P2: Female, 35-44 years, NZ European

Several reported a desire to keep check-ins as part of usual practice or identified that telehealth services could be an in-between service or a way to extend what was seen as usual in-person care.

If there was the possibility of getting more services, more talk time with the doctor, if the service could be extended via Zoom, by online meetings.

P1: Male, 35-44 years, NZ European

Few reported having videoconferencing appointments and suggested that inadequate resourcing of mental health teams contributed to the types of appointments offered.

It was just a resourcing thing [to not offer Zoom]… I know that they’re not greatly funded… do we have enough internet to be able to cope with this and do we need an extra package to pay for the bandwidth for these doctors doing it, do we have cameras, do our clients or patients have the cameras? To have the actual Zoom… you have to pay… It probably became a too big problem to sort out.

P14: Female, 35-44 years, mixed ethnicity American

Technology often needed to be customized to client requirements (predominantly a technological capability issue, rather than mental health needs), diverting attention in appointments from clinical to technological matters. In turn, a perceived lack of clinician technological skill meant that clients felt stressed and responsible for making technicalities related to telehealth work to access needed services. This issue reflected a primary recommendation from several participants to improve clinician telehealth literacy.

The psychiatrist and [care worker]… left it up to me to make a new [Zoom] meeting room and I had to do it. I was like, this is not my job. I shouldn’t be bloody doing it.

P11: Male, 35-44 years, NZ European

### Maintaining and Building Relationships

Many participants worked with clinicians with whom they had longstanding trusting relationships. This helped with transitioning to a telehealth service and meant that participants felt clinicians would put effort into maintaining contact. Most advised that an established relationship was a prerequisite for an effective telehealth service.

To trust them, you have to see them a couple of times and get to know them.

P10: Male, 65+ years, NZ European

Some participants were concerned that under telehealth, the lack of a personal element could negatively affect interactions or make it difficult to start relationships with clinicians. Participants suggested that poor initial relationships with clinicians could be intensified in telehealth services.

It just makes things more personal, and you can develop that relationship with a person better if you can see them.

P1: Male, 35-44 years, NZ European

Participants spoke about difficulties in maintaining relationships when contact arrangements became depersonalized. One example of this related to receiving telephone calls from mental health services. Mental health services blocked caller identification so clients could not respond to missed calls. One participant when talking about telephone calls described this as follows:

They would say they couldn’t get hold of me. I’d say, well, have you left a message? No. Well, how can I call you back?… It’s the biggest frustration when you get nine missed calls from no caller ID. Great, someone really wants to get hold of me, but I don’t know who, and I don’t know how urgent it is.

P5s: Male, 35-44 years, NZ European

However, established relationships were not seen as important for everyone. For some participants newer to the service, telehealth was seen as an opportunity to develop trust in clinicians while remaining within a safe home-based environment. One support person, when speaking about videoconferencing appointments, noted the following:

He was at home in his safe place, he could build up a rapport with [the clinician] from the beginning before he actually met him face to face in real life, so it was really good from his perspective. It was nice and relaxed.

P17s: Female, 45-54 years, NZ European

Other participants suggested that because clinicians were also based at home, telehealth services provided clients with the opportunity to glimpse clinicians’ personal lives and build individual relationships. In relation to videoconferencing, 1 participant noted:

You could see most of her lounge… I said, “I like that, can I have it?”. She said, “Yeah, when I die”... She made a joke out of them. I thought that was pretty cool.

P11: Male, 35-44 years, NZ European

### Discerning Nonverbal Cues: Interpreting the Gaps in Conversation

Participants identified that telehealth appointments, particularly telephone-based appointments could lead clients and clinicians to miss important nonverbal cues. Participants suggested that for clinicians, these cues could relate to such things as clients shaking, changes in alcohol consumption, or other behavioral changes that could be hidden if not in the same room with the clinician.

Often when I’m stressed out, I will be shaking, or something like that. Those little intricacies, or lack of eye contact, all those things that they look at to determine how well somebody is. You’re missing that when you’re on the phone.

P14: Female, 35-44 years, mixed ethnicity American

Some participants also perceived that receiving telehealth services meant clients lacked cues to support their engagement in consultations. For example, those with memory issues (or their support people) indicated that they lacked visual anchors to support their retention of information, rapport building, or engagement in consultations. Others highlighted that being unable to see their clinicians’ body language could create uncertainty. This occurred because clients could not be sure that their clinicians were listening, or as 1 participant described:

It was easy to ring them but that’s not the sort of contact that I want. I want a real person in front of me talking. It’s more reassuring… It’s just more reassuring knowing that the person’s there for you, and not pulling faces on the phone trying to hang up on you… because that’s what they always try to do to me.

P7: Female, 35-44 years, Māori

Telehealth provided additional time and space for clients to mask distress. The same participant explained that in-person care created a second set of eyes to monitor risky behavior.

You [health care professionals] are like second eyes… You can see everything first-hand. So if you saw,… a beer at seven o’clock in the morning, you’ll be like, what are you doing drinking?... And then we could nail the problem straight away… If you don’t snap me in action then, I’m a keep going.

P7: Female, 35-44 years, Māori

In contrast, another participant, who communicated with their clinicians via email, suggested that this media gave them time to express themselves in a more controlled manner:

It’s easier for me to converse [via email] because I have time to think about what I want to say as opposed to just talking and not thinking about what I say.

P9: Female, 55-64 years, NZ European

### Creating Safe Spaces and Involving Others

Participants spoke of how receiving mental health support while at home changed their engagement with care based on safety perceptions. Telehealth offered a distinct flexibility advantage in that support people could be involved in part of an appointment without staying for the whole session. In addition, participants described their homes as being less confronting, busy, or stressful than a clinician’s office. They suggested that home environments provided clients with access to personal comforts and the ability to speak freely on difficult topics while retaining personal space. One participant, who described telephone-based therapy as normal for them, suggested the following:

I usually don’t talk in real life because that’s a bit too overwhelming for me… but when I’m in my house, I’m loose and would just talk freely.

P8: Female, 18-25 years, Māori

However, other participants indicated that receiving services in their homes led them to confront personal issues on a videoconferencing or telephone platform that they felt was more appropriate for socializing. Participants also discussed not having access to sensory equipment (such as rocking chairs, sand, and toys) that may have facilitated engagement with treatment and supported feelings of calm during appointments. Some participants noted that having family around or living close to others (eg, neighbors who could look through windows) made telehealth consultations less private. In turn, this may have contributed to some participants feeling they could (1) manage their mental health needs without the need for clinical input or (2) withhold information. However, not all participants identified these consequences, in part, perhaps, due to the strong relationships between clinician and client.

My 11-year-old wouldn’t just let me go in a room and be; she’d be listening in at the door or something at the least. Also, I couldn’t have really said anything because my kids were there.

P2: Female, 35-44 years, NZ European

Other participants discussed their strategies to ensure privacy, for example, setting up booking processes for bedrooms in their homes, moving to quiet places within their house, and closing curtains. Confidentiality of the medium was a key factor for some, with differences in how various forms of telehealth were perceived in terms of privacy.

I don’t know enough about Zoom to know in her [the clinician’s] particular area how confidential Zoom is... I don’t know whether you can go back and replay a Zoom meeting... With a phone, you hang up, it’s finished.

P10s: Female, 65+ years, NZ European

Participants were generally comfortable with their clinicians working from home. They indicated that consultations were less likely to be interrupted by external factors such as the next client, or a telephone ringing, and suggested that clinician’s being in a safe space influenced appointment success.

It [telehealth] was nice for [my child] as well, because it’s not sort of like here, you've got four stark walls kind of thing with posters to do with mental health and stuff, so I think that probably helped relax him as well, because [the clinician] was in a relaxed space and situation.

P17s: Female, 45-54 years, NZ European

However, participants also voiced concerns about clinician privacy in home environments. Participants were worried that there would be other people listening to private conversations with their clinician, such as family or other clinicians. They were uncomfortable discussing sensitive issues, such as trauma. Furthermore, participants were uncomfortable discussing privacy or were unaware of how to approach this with clinicians.

I have no idea what her end was like. I didn’t know who was around. I didn’t know where she was… It’s kind of stressful to ask where are you, who’s around?

P4: Gender diverse, 18-25 years, Māori, New Zealand European

### Collaboration and Clinician Readiness

Participants showed great awareness of how service coordination and integration affected their health care journeys. They suggested that lockdown may have enabled improvements in collaboration between clinicians, as they would have practiced more deliberately and without incidental opportunities for communication. Conversely, for other participants, coordination between clinicians worsened.

I was receiving phone calls from four or five different agencies about slightly different things, but it gets very confusing about which group has influence over which things… And they’re all phoning me. They don’t know I’ve spoken to the other groups, which I find really strange.

P5s: Male, 35-44 years, NZ European

At times, systems established for pre-lockdown environments failed to work in lockdown conditions, as these systems did not consider the impact of a pandemic on service accessibility.

I said, “Well you know you’re actually putting me in more danger just because I’ve missed appointments [because of lockdown isolation] that I have no control over…, now I’m having to go to the afterhours [medical centre to pick up my methadone] where there’s actually a COVID testing station. For the sake of your bureaucratic little system, you’re endangering me.”

P13s: Male, 35-44 years, NZ European

Organizational issues impacted clients, they felt forced to take the initiative for getting their required care, be this through setting up appointments or liaising between care providers. However, even when trying to access services, pandemic-related changes led to a lack of coordination.

### Having Options

Most participants, regardless of preference for in-person or telehealth services, were in favor of a flexible service allowing clients to choose their best delivery mechanism. Some suggested that a flexible telehealth service could meet client needs on bad days, or if clients were uncomfortable or unable to attend in-person outpatient appointments. Moreover, telehealth was seen as a way for clients to get more time and more frequent appointments with their clinician over and above what could normally be possible with in-person care.

Give the option of “we can do a Zoom”, “you can come to me”, “how would you like to do it?”. That gives the person who’s going to the service that little bit more of control.

P17s: Female, 45-54 years, NZ European

When considering the need for options, however, several participants described preferences for videoconferencing for personal or family conversations but not for clinical appointments. Telehealth was generally not seen as supporting the culture of clinical appointments. One participant made the point that while people may be used to communicating through social media and other telecommunication applications, using telecommunications technology to treat people is still a new practice.

They’re thinking in terms of well, I’ve rung up the grocery to get some bread,… now I’ll ring up the patient to get whatever… They think this phone call is a pretty ordinary thing that’s been going on for the last 80 years… but we’re not used to it in any way under this heading [delivering mental health services].

P10: Male, 65+ years, NZ European

Participants presented areas for consideration when developing telehealth-based services. For many, the benefit of telehealth was that they did not have to put as much time into appointments, including, for example, travel time. Some suggested that while telehealth may be a good option, the specific needs of the mental health population should determine how it is used. Key considerations included client (1) engagement with care, (2) access to sensory therapy items, (3) ability to advocate for preferred services, and (4) memory and hearing issues. For example, telehealth-based services were not necessarily appropriate for Deaf or hearing-impaired people because synchronization problems between video and audio in videoconferencing complicated lipreading.

I need to be able to lipread. I tried the video, I tried putting my headphones on. I can hear you, but I need to be able to read lips. But when you see people on the video, they talk a bit then the video… what’s that word [it goes out of sync]?

P11: Male, 35-44 years, NZ European

## Discussion

### Principal Results

Out of necessity, the COVID-19 pandemic caused abrupt and dramatic shifts toward telehealth-based mental health services. Our study explored the views of 21 mental health clients and the people who support them. Participants highlighted key themes characterizing telehealth experiences, these are (1) changes to service delivered by telehealth, (2) maintaining and building relationships; (3) discerning nonverbal cues; (4) creating safe spaces and involving others; (5) collaboration and clinician readiness; and (6) having options.

### Comparison With Prior Work

Participants highlighted the importance of preexisting relationships with their named clinician to enable the effective delivery of telehealth services. Such findings build on those in primary health care and within mental health that suggest that existing relationships enable service users to feel more at ease [26,27]. Participants expressed concern that client-clinician relationships would be difficult to establish over telehealth, although 1 participant did find it possible. Telehealth service delivery may influence relationships between client and clinician as clients learn about aspects of clinician life previously hidden, consistent with findings from our research into clinician perspectives [28]. Telehealth adoption may negatively affect clinical practice and therapeutic relationships [29]; this change in relationships also has the distinct advantage of humanizing interactions about mental health, and thereby removing some of the sterility of in-person outpatient appointments. As such, it is paramount to identify design features within a telehealth consult that promote engagement and therapeutic relationship building, particularly given recent research suggesting that therapeutic alliances are weaker when services are delivered online [30,31]. On top of this, a lack of familiarity with telehealth within New Zealand’s mental health services also points to the value of culture change to embed telehealth as a service delivery option.

Overall, there is a need to scope the purpose of telehealth appointments better; clients and clinicians should agree whether each telehealth appointment is intended to act as a check-in between in-person appointments or as a clinical service. Such agreements should be regularly reviewed and could at least be reviewed when orientating clients to new services and when reviewing clients’ treatment goals. Adjusting the scope of telehealth accordingly could improve client outcomes, trust, and service engagement in a medium that does not necessarily fit the culture of clinical (in-person) appointments but that could be an adjunct service delivery option. This suggestion has also been made by other researchers who highlight client preference for telehealth to be used flexibly as an alternative or addition to in-person treatment [9,14,18,27,32,33]. In this way, telehealth should not simply be considered a different platform but a different way of working [27]. Significantly, setting unambiguous scopes around telehealth appointments may also reduce the blurring of boundaries research participants raised in this and other studies [28] when clinicians work from home.

Research participants suggested that mental health services lacked organizational readiness to deliver care via telehealth, with limitations in service collaboration and system compatibility, and clients and clinicians who lacked technological knowledge. Strengths included a change to more proactive communication between clinicians. Dedicated proactive support is needed for clients and clinicians to ensure their technological proficiency and their access to necessary resources. For clinicians, we echo suggestions from other researchers [14,18,34,35] that there is a need for increased access to training and professional guidance from health professional representative bodies. A common (mental health) information technology platform and a strategy for the effective use of telehealth across services could support the evolution of business-as-usual practice to include telehealth as a workable option.

### Recommendations for Practice

To deliver effective telehealth services, it is imperative that client voices are included in policy and planning around the use of telehealth and as part of wider service delivery. Mental health teams should have standardized policies on appointment confidentiality, information recording, and involvement of others in appointments. For individual clients, a key step to care continuity (whether this be via telehealth or in-person) is to ensure the integration of services so that clients are not responsible for connecting different service delivery teams. Policy standards should be created with clients and consistently communicated to them, perhaps through information in a telecommunication application–based waiting room. Responsibility for upholding these standards should be clearly placed with treating teams and monitored for compliance. This is essential to ensuring telehealth services meet an appropriate standard that fits the intent behind New Zealand’s 1996 Code of Health and Disability Services Consumers' Rights and arguably expands the original remit of this code.

When conducting our interviews, it became apparent that some participants had difficulty consistently articulating their views. Furthermore, participants had reservations about (1) retaining information provided to them via telehealth, (2) clinicians and clients picking up nonverbal cues (such as tremors, the aroma of alcohol, and other psychomotor changes), and (3) being able to communicate if hearing impaired or Deaf. Nonverbal cues are important for establishing trust, exchanging information, and for the clients to get feedback on a clinician’s response to them, an issue others have also raised [36,37]. Effective communication is core to truly embedding person-centered care into practice [38]. Our findings concerning hearing impairment mirror those of earlier research into Deaf people’s experiences of health care access over the pandemic in New Zealand [39]. While research into telehealth has largely suggested the equivalence of this delivery method, telehealth could allow some clients to be underserved, specifically, those who are less proficient at verbalizing their distress; this could also affect a client’s willingness to use telehealth. In addition, when moving toward further digital-based services, acknowledging and acting on changes in power distribution between client and clinician is essential to ensuring moves toward person-centered care [40]. Key practical steps clinicians and mental health services should consider when using telehealth are laid out in Textbox 1.

**Practical telehealth considerations when working with mental health clients.**

**Responsibility: clinician**

- Recognize that telehealth consultations may not serve the same function as in-person consultations.
- Work with clients to identify the types and frequency of consultations that are suitable for telehealth. This should also include identifying when telehealth appointments are inappropriate.
- Set parameters for telehealth appointments, including around privacy (recording of appointments and attendees) and where appointments will be held.
- Work with clients to identify and document warning signs clinicians should watch for in telehealth appointments.
- Work with clients to identify preferred alternative clinicians if clients cannot receive services from their preferred provider.

**Responsibility: outpatient service and wider health sector**

- Ensure that clinicians are trained in (1) how to conduct telehealth-based appointments and (2) how to conduct telehealth-based mental health assessments.
- Provide clinicians with resources to create spaces within their home or outpatient team to ensure their privacy during appointments.
- Work with clinicians and clients to establish procedures for informing clients about how services will be delivered should there be a sudden shift to telehealth-based care.
- Ensure that client files are current, including information on all health and social services with whom clients interact; this is core to ensuring the integration of disparate health care organizations.
- Fund and maintain telehealth-capable systems, including resourcing computers and ensuring access to appropriate videoconferencing platforms.

### Limitations and Future Research

As with any qualitative study, generalizability is not the aim; limitations to sample size and diversity further limit the potential generalizability of findings. Rather, in drawing attention to participant perspectives, our research generates valuable recommendations for how telehealth-based mental health services can better meet client needs during routine and pandemic situations. Alongside our research into clinician perspectives [28], this work is important for ensuring that client experiences are at the front of any telehealth service planning decision, particularly in light of the announced Royal Commission of Inquiry into New Zealand’s COVID-19 response [41]. Further, information on the mental health diagnosis of research participants has not been provided because of concerns about identifying them. Having this information may have allowed more insight into the influence of diagnosis on participant experience and use of telehealth, particularly given that participants represented those with particularly high service needs.

This research could be strengthened through a separate project investigating the ongoing therapeutic engagement with mental health services following a return to usual in-person care. As part of this, identifying themes common to building and maintaining relationships within a postacute COVID-19 health care environment will be crucial to any attempt to reintroduce telehealth to mainstream mental health care. Additional research is also needed into the effects loss of nonverbal cues in telehealth has on client outcomes, therapeutic relationships, and client confidence, particularly for those less able to verbalize their requirements.

### Conclusions

The COVID-19 pandemic prompted unprecedented changes in mental health outpatient service delivery and temporarily introduced telehealth into mainstream practice. Yet, services delivered via telehealth differed from in-person care and require consideration of how to manage relationships, discern nonverbal cues, and create safe spaces. Key to effective telehealth delivery is safeguarding minimum practice standards related to telehealth fit with clinical needs and service readiness. A tangible first step in this process is to ensure that upon orientating clients to new services and when reviewing clients’ treatment goals, client-specific procedures around telehealth use are updated, and protocols maintained in client notes. In turn, health systems must ensure that clinicians access training and have professional guidance on how to deliver effective telehealth consultations. This should help in ensuring telehealth services uphold a minimum standard of care and clients’ rights to appropriate services.

## Appendix

Multimedia Appendix 1 Interview schedule.

Multimedia Appendix 2 Standards for Reporting Qualitative Research (SRQR) checklist.

## Data Availability

Data sharing is not applicable to this article as no data sets were generated or analyzed during this study.
