# Supplementary material for: Mental Health Client Experiences of Telehealth in Aotearoa New Zealand During the COVID-19 Pandemic: Lessons and Implications
Source: JMIR Form Res. 2023 May 26;7:e47008. doi: 10.2196/47008 (PMC10257105; doi:10.2196/47008)
Supplement: Multimedia Appendix 1 [file formative_v7i1e47008_app1.docx]

| 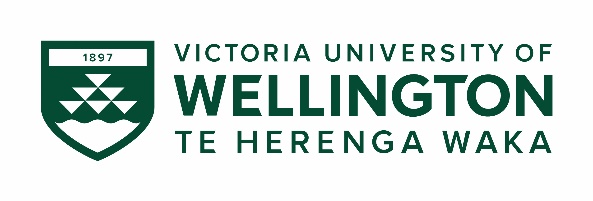 | Telehealth, protect the bubble: mental health service delivery during COVID-19 |
| --- | --- |

## Interview schedule clients

Introduction: This research study will be looking at how telehealth was helpful or not in delivering mental health outpatient services during alert levels 3 and 4 of COVID-19. When we say the word telehealth, we’re thinking of video conferences/ hui (like those via Zoom) and telephone consultations. We are especially interested in topics such as how you perceived service offered in this way, what things you would like to see changed, and how telehealth worked in practice*. We are also interested in gaining an understanding of how you saw overall service delivery change as a result of using telehealth to deliver care during alert levels 3 and 4.

Everything you say today is confidential, which means I will not directly attribute anything to you, nor will MHAIDS know that you have chosen to participate in an interview. It’s your choice whether you answer questions, you can choose to stop at any time, ask for your answers to not be recorded, or for the recorder to be paused at any time. Feel free to ask questions throughout, but before we start, do you have any questions?

**where parents/ guardians are speaking on behalf of a client under 18 years, the questions will relate to the treatment the client received.*

1. How did you find lockdown?

| Prompts: | During the COVID-19 pandemic did you make any changes to how you usually manage your health or care for yourself? These could be positive or negative changes, for example, changes to diet, exercise, medicine/ alcohol taking, pharmacy services? What? Why? |
| --- | --- |

1. What were your expectations around receiving mental health services during COVID-19 alert levels 3 and 4?
2. What MHAIDS clinic/ provider do you usually go to?
3. How easy (or not) was it to get in contact with MHAIDS/ arrange services over alert levels 3 and 4? Did this differ from usual?
4. What were your expectations around receiving telehealth services as provided by MHAIDS during alert levels 3 and 4?
5. Can you tell me about your experience with receiving services through telehealth during alert levels 3 and 4?

| Prompts: | How often? Was this more/ less frequently than before? |
| --- | --- |
|  | Who with? Did you find telehealth more effective with different types of clinicians? Why/ why not? |
|  | What form? (telephone, video conferencing e.g. Zoom) |
|  | How did it go? How was the quality? |
|  | How long were appointments? Was this more/ less than before? |
|  | How was it initiated e.g. were you provided with information on how to use Zoom/ how the consultation would run? |
|  | What worked well? |
|  | What didn’t work well? (e.g. responsiveness, wrap-around services, prescription access) |
|  | What technical/ IT difficulties arose when using Zoom/ other form of telehealth and how were they overcome/ what IT support was available to you? |
|  | How did you maintain your privacy when using telehealth? e.g. family coming into the room, being overheard etc. |
|  | Did you access other telehealth services outside of MHAIDS? |
|  | Before receiving services in this way over COVID-19, had you received telehealth services before? |

1. How does the service provided via telehealth differ to what you receive as part of usual care?

| Prompts: | Cultural responsiveness/ safety |
| --- | --- |
|  | Whānau involvement |
|  | Timeliness |
|  | Accessibility |

1. How did receiving services in this way (via telehealth) influence your ability to manage your own care?

| Prompts: | How did it influence your relationship with your clinicians? e.g. ease of access, timeliness, ability to talk with them, record conversations |
| --- | --- |

1. How did receiving services in this way (via telehealth) influence your clinicians’ ability to manage your care?

| Prompts: | How did it influence the collaboration between your different clinicians? |
| --- | --- |
|  | How did it influence the clinician’s ability to follow up with you? |
|  | How did it influence the type of therapy/ service offered? |
|  | Was MHAIDS ‘ready’ to use telehealth, why/ why not? |

1. What elements were important to ensuring the success of telehealth consultations for you?
2. What elements do you think are important to ensuring the success of telehealth for your care team/ service provider/ individual clinician?
3. Has any component of the telehealth service delivery model changed the way you’ve felt about your own condition? What component? How?
4. What were the outcomes of the telehealth interaction, did you get what you needed?
5. Were you able to have whānau/family involved in telehealth consultations, and how would you want whānau/family to be involved in these consultations?
6. Non face-to-face consult methods such as telephone or video consults may work better for some people than others – and better for some health problems than others. In what circumstances do you think non face-to-face consults would work well for you, and when do you think they wouldn’t work so well?

| Prompts: | When could kanohi ki te kanohi (face to face) work better? |
| --- | --- |

1. What elements of telehealth service delivery would you like to see kept as part of the usual services provided? Why/ why not?
2. Many thanks for your time today, before we finish, is there anything else you would like to add?
3. Ask to fill in demographic form if not already completed.

**Support resources if clients become distressed**

Healthline 0800 611 116

COVID Healthline 0800 358 5453

General mental health support

<https://www.depression.org.nz/>

<https://www.allright.org.nz/>

<https://www.thelowdown.co.nz/>

<https://www.auntydee.co.nz/tips-and-help> (mental health resource for Pacific people)
